# Panoramic view of clinical features of lupus erythematosus: a cross-sectional multicentre study from China

**DOI:** 10.1136/lupus-2022-000819

**Published:** 2023-03-20

**Authors:** Hui Jin, Shihang Zhou, Yangyiyi Yu, Ming Zhao, Haijing Wu, Hai Long, Siqi Fu, Ruifang Wu, Heng Yin, Jieyue Liao, Shuangyan Luo, Yu Liu, Qing Zhang, Peng Zhang, Yixin Tan, Shuaihantian Luo, Xin Huang, Fen Li, Guanghui Ling, Qianjin Lu

**Affiliations:** 1 Department of Dermatology, The Second Xiangya Hospital of Central South University, Changsha, China; 2 Research Unit of Key Technologies of Immune-related Skin Diseases Diagnosis and Treatment, Chinese Academy of Medical Sciences Institute of Dermatology, Nanjing, China; 3 Institute of Dermatology, Chinese Academy of Medical Sciences and Peking Union Medical College, Nanjing, China; 4 Key Laboratory of Basic and Translational Research on Immune-Mediated Skin Diseases, Chinese Academy of Medical Sciences, Nanjing, China; 5 Jiangsu Key Laboratory of Molecular Biology for Skin Diseases and STIs, Nanjing, China; 6 Hunan Key Laboratory of Medical Epigenomics, The Second Xiangya Hospital of Central South University, Changsha, China; 7 Department of Rheumatology and Immunology, The Second Xiangya Hospital of Central South University, Changsha, China

**Keywords:** lupus erythematosus, systemic, lupus nephritis, autoimmune diseases

## Abstract

**Objective:**

Lupus erythematosus (LE) is a complicated disease with highly heterogeneous clinical manifestations. Previous studies have rarely included all subgroups of patients with lupus and have overlooked the importance of the cutaneous manifestations thereof. We aimed to compare the demographic and clinical differences among patients with different subtypes of lupus.

**Methods:**

This is the first real-world study with a relatively large sample size that simultaneously includes patients with isolated cutaneous lupus erythematosus (iCLE) and SLE. All samples were obtained from the Lupus Erythematosus Multicenter Case–control Study in Chinese populations (LEMCSC) (registration number: ChiCTR2100048939). Comparative analyses between different LE subgroups were performed.

**Results:**

A total of 2097 patients with lupus were included, with 1865 patients with SLE, 1648 with cutaneous lupus erythematosus (CLE), and 232 with iCLE. Among the patients with CLE, 1330 had acute cutaneous lupus erythematosus (ACLE); 160 had subacute cutaneous lupus erythematosus (SCLE); and 546 had chronic cutaneous lupus erythematosus (CCLE). The study included a relatively large number of patients with CCLE subtypes, including 311 with discoid lupus erythematosus (DLE), 262 with chilblain lupus erythematosus (CHLE) and 45 with lupus erythematosus profundus (LEP). Demographic characteristics, systemic involvement, mucocutaneous manifestations and autoantibodies were significantly different among the groups.

**Conclusions:**

CLE and iCLE are two distinct disease states, and the selection of broad or narrow CLE definitions should be emphasised in scientific reports. LE-non-specific cutaneous lesions imply more severity, while self-reported photosensitivity and LE-specific cutaneous manifestations imply milder severity. Generalised ACLE appears to be a more severe state than localised ACLE, and CHLE appears to be more severe than DLE. Anti-Sjögren’s syndrome-related antigen B (SSB) antibodies have higher specific directivity than anti-Sjögren’s syndrome-related antigen A (SSA) antibodies for SCLE lesions. Anti-double-stranded DNA antibodies have a higher co-occurrence with ACLE and a lower co-occurrence with SCLE and CCLE. Compared with DLE, CHLE has significantly higher positive rates of anti-SSA/Ro60 (71%) and anti-SSA/Ro52 (42.4%) antibodies, whereas LEP is associated with a higher positive rate of antinucleosome antibodies (31.1%).

**WHAT IS ALREADY KNOWN ON THIS TOPIC**

- Lupus erythematosus (LE) is a broad spectrum of disease that includes a variety of subtypes, including SLE, cutaneous lupus erythematosus (CLE) and isolated cutaneous lupus erythematosus (iCLE), and different subgroups with unique features; however, previous studies lacked large-sample studies covering all subtypes of lupus.

**WHAT THIS STUDY ADDS**

- This is the first real-world study with a relatively large sample size that simultaneously included patients with SLE, CLE and iCLE to provide a panorama of the demographic and clinical characteristics of patients with different LE subtypes: LE-non-specific cutaneous lesions imply more severe severity, while self-reported photosensitivity and LE-specific cutaneous manifestations imply milder severity; generalised acute cutaneous lupus erythematosus (ACLE) appears to be more severe state than localised ACLE, and chilblain lupus erythematosus (CHLE) appears to be more severe than discoid lupus erythematosus (DLE); anti-Sjögren’s syndrome-related antigen B (SSB) antibodies have higher specific directivity than anti-Sjögren’s syndrome-related antigen A (SSA) antibodies for subacute CLE lesions. Compared with DLE, CHLE has significantly higher positive rates of anti-SSA/Ro60 (71%) and anti-SSA/Ro52 (42.4%) antibodies, whereas lupus erythematosus profundus is associated with a higher positive rate of antinucleosome antibodies (31.1%).

**HOW THIS STUDY MIGHT AFFECT RESEARCH, PRACTICE OR POLICY**

- The LE clinical panorama promotes stratified management, improves economic efficiency and may improve prognoses.

## Introduction

Lupus erythematosus (LE) is a complicated disease with highly heterogeneous clinical manifestations. Its heterogeneity is not only reflected in the fact that SLE can affect any organ of the human body,^1^ but also in the complex manifestations in the cutaneous system, which can be classified into LE-specific cutaneous manifestations and LE-non-specific cutaneous manifestations. LE-specific cutaneous manifestations, also known as cutaneous lupus erythematosus (CLE), can be treated as diagnostic clues, as they are not observed in other disorders. In contrast, LE-non-specific cutaneous manifestations are related to LE but are not specific.^2^ The Core Set Questionnaire developed by the European Society of Cutaneous Lupus Erythematosus (EUSCLE) provides a comprehensive assessment method of mucocutaneous involvement in lupus.^3^ According to the Duesseldorf Classification, CLE is classified into four major categories: acute cutaneous lupus erythematosus (ACLE), subacute cutaneous lupus erythematosus (SCLE), chronic cutaneous lupus erythematosus (CCLE) and intermittent CLE (ICLE).^3–5^ The major categories of CLE can also be further grouped into several secondary subtypes: ACLE consists of localised and generalised forms; SCLE, annular and papulosquamous types; CCLE, discoid lupus erythematosus (DLE); lupus erythematosus profundus (LEP); and chilblain lupus erythematosus (CHLE).^4 6^

LE-non-specific cutaneous manifestations include photosensitivity, oral ulcers, non-cicatricial alopecia, Raynaud’s phenomenon, vasculitis, etc.^3 4 7^ In the EUSCLE Core Set Questionnaire, photosensitivity is self-reported by the patient based on history, whereas other skin lesions are objectively present.^3^ Therefore, we define LE-non-specific cutaneous manifestations other than photosensitivity as LE-non-specific cutaneous lesions to distinguish them from photosensitivity.

There is no clear boundary between SLE and CLE in the lupus spectrum. A total of 5%–25% of patients with pure mucocutaneous manifestations can develop SLE, and up to 80% of patients with SLE have cutaneous manifestations.^4 7–9^ The definition of CLE remains controversial. Traditional CLE refers to patients with only LE-specific cutaneous manifestations, excluding a diagnosis of SLE.^4^ This is a narrow definition, and several previous epidemiological investigations of CLE have been based on this definition.^10^ Another definition of CLE is equivalent to LE-specific cutaneous manifestations, regardless of whether they are accompanied by systemic involvement.^11^ This concept is more beneficial to the clinical diagnosis and scientific research of patients with lupus, since up to 50% of patients with SCLE meet the criteria for SLE, and most patients with ACLE have systemic involvement.^4 7 8^ Many previous studies have adopted this broad definition of CLE,^11^ as we did in the present study, referring to the narrow definition of CLE as pure CLE^12^ or isolated cutaneous lupus erythematosus (iCLE).^10^

Different cutaneous manifestations can provide clues to disease evolution and prognosis.^4 7^ Most previous studies on SLE only describe the four cutaneous manifestations involved in the American College of Rheumatology (ACR) SLE classification criteria (malar rash, discoid rash, photosensitivity and oral ulcers).^13–15^ Focusing on only these items is far from sufficient. Thus, there is an urgent need to investigate the cutaneous manifestations of SLE in detail. In addition, previous studies have rarely included all subgroups of patients with lupus, while the sample sizes of most previous studies regarding clinical characteristics of CLE/iCLE are relatively small.^16 17^ Most studies have focused on a single category of SLE or CLE, rather than both. A European cross-sectional study of 1002 patients with CLE provided a comprehensive mucocutaneous description of lupus based on the EUSCLE.^11^ However, this study did not include patients with SLE without LE-specific cutaneous manifestations; therefore, it is impossible to compare the differences between patients with LE with or without LE-specific cutaneous manifestations.

We aimed to determine the differences in the clinical characteristics of the different subtypes of lupus, the clinical panorama of LE and the relationship between complex mucocutaneous manifestations and systemic involvement by investigating the data from 2097 Chinese patients with LE. This is the first real-world study with a relatively large sample size that simultaneously included patients with iCLE and SLE.

## Methods

### Study design

The study was based on the Lupus Erythematosus Multicenter Case–control Study in Chinese populations (LEMCSC) (registration number: ChiCTR2100048939).^18 19^ From December 2013 to December 2015, the LEMCSC recruited patients with LE from inpatients and outpatients of the dermatology department or inpatients of the rheumatology/nephrology department in 29 hospitals (30 centres) in 15 provinces in China.

### Study population

To fully cover all subtypes of LE, patients who met at least one of the following criteria were enrolled: (1) patients who fulfilled at least one of the 1997 American College of Rheumatology (ACR97)^20^ and the 2012 Systemic Lupus International Collaborating Clinics (SLICC12)^21^ SLE classification criteria (most of these patients came from the rheumatism and nephrology wards) and (2) patients with any LE-specific cutaneous manifestations (most of these patients came from dermatology clinics or wards). Patients who refused informed consent or were unable to cooperate with the normal assessment due to physiological defects (such as blindness and deafness) or critical illness were excluded.

### Variables

The collected variables included demographic characteristics, systemic involvement, mucocutaneous manifestations and laboratory test results (autoantibodies). Systemic lupus involvement (arthritis, renal involvement, haematological abnormalities, serositis and neurological involvement) was defined according to either the ACR97 or SLICC12 criteria. Mucocutaneous manifestations (LE-specific cutaneous manifestations and LE-non-specific cutaneous manifestations) were assessed using the Chinese version of the EUSCLE Core Set Questionnaire.^3 11^ Autoantibodies were tested at the clinical laboratory of each hospital, which were all tertiary grade A hospitals with internationally recognised reagents and stable negative control methods.

### Data collection

Written informed consent was obtained from all patients. After on-site and online training organised by the Command Centre of the Second Xiangya Hospital of China, assessors collected data using a paper form according to a unified standard. Each patient was assessed face-to-face by at least one highly trained dermatologist to ensure comprehensive collection of cutaneous manifestations. Through face-to-face interviews and medical record reviews, we collected clinical characteristics at three time points: onset, most severe and investigation.

The evaluators recorded LE-related mucocutaneous manifestations according to a standardised evaluation manual, developed based primarily on the definitions recommended by the EUSCLE Core Set Questionnaire with a large number of sample pictures from Chinese patients with lupus. The judgement of each CLE subtype was based on the expert consensus of Kuhn and Landmann, who developed the EUSCLE Core Set Questionnaire.^5^ The definition of each subtype of CLE is consistent with the international consensus published later,^4 22 23^ which was the result of comprehensive considerations of lesions, autoantibodies and skin pathology. For patients with typical clinical manifestations and consistent autoantibodies, skin biopsy was not necessary.

In this study, 36.7% of the patients with CLE underwent skin biopsy; most had negative autoantibodies or atypical clinical manifestations. Patients with suspected CLE with atypical clinical manifestations and no pathological results were excluded. The central unit set up an expert consultation group, and researchers in each subcentre could share photos and initiate consultations if they had any questions during data collection. The expert consultation group was required to provide feedback within 24 hours to ensure the accuracy of the original records.

**Figure 1:**
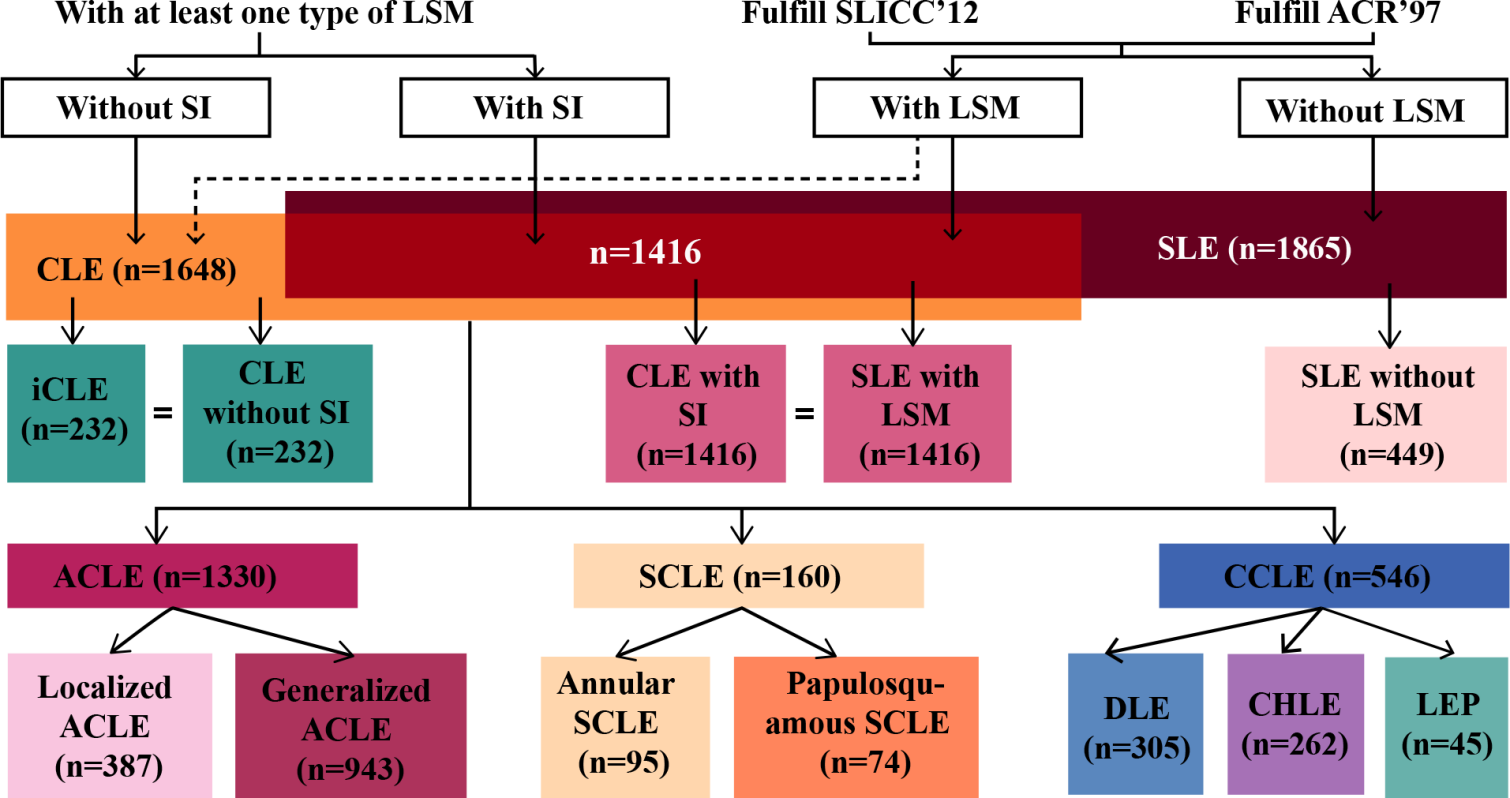
Flowchart of sample inclusion and grouping. ACLE, acute cutaneous lupus erythematosus; ACR97, 1997 American College of Rheumatology; CCLE, chronic cutaneous lupus erythematosus; CHLE, chilblain lupus erythematosus; CLE, cutaneous lupus erythematosus; DLE, discoid lupus erythematosus; iCLE, isolated cutaneous lupus erythematosus; LEP, lupus erythematosus profundus; LSM, lupus erythematosus-specific cutaneous manifestation; SCLE, subacute cutaneous lupus erythematosus; SI, systemic involvement; SLICC12, 2012 Systemic Lupus International Collaborating Clinics.

All paper collection forms were mailed back to the command centre and, after a quality audit to check completeness, eligible forms were independently reviewed by two dermatologists. In combination with clinical manifestations, laboratory examinations and histopathological features, the final diagnosis and grouping of each patient were confirmed if the two dermatologists had the same grouping results. Otherwise, the paper form was submitted to a panel of three dermatologists and two rheumatologists for further discussion, and the final diagnosis was obtained when three or more experts reached a consensus. The paper materials were inputted electronically by 32 undergraduate volunteers through a double-record verification strategy using EpiData V.3.1, as per our previous study.^18^ Figure 1 shows the grouping and analysis process. ICLE was not analysed in this study because of the lack of samples.

### Statistical analysis

To comprehensively present the characteristics of lupus, this analysis used the data of each patient throughout the course, which included the accumulation of data at the onset, the most severe and the investigation. A positive result at any of the three time points was considered positive throughout the course. Data analysis was performed using R software V.4.1.2. Non-normally distributed continuous variables are expressed as median (Q1, Q3). Categorical variables are expressed as frequencies with percentages (%). The Kruskal-Wallis test was applied to samples with non-normal distribution to compare the population distribution. The χ^2^ test or Fisher’s exact test was used to analyse categorical variables. The independent variables of the multivariate logistic regression were selected based on clinical experience, assisted by the least absolute shrinkage and selection operator (LASSO) regression analysis. We then calculated the adjusted OR, 95% CI and P value using Wald’s test. A p value of <0.05 was considered statistically significant and was corrected for multiple comparisons using Bonferroni correction.

### Patient and public involvement

Patients and the public were not involved in the design, conduct, reporting or dissemination plans of our research.

## Results

### Overall demographic and clinical features in patients with SLE, CLE and iCLE

**Figure 2:**
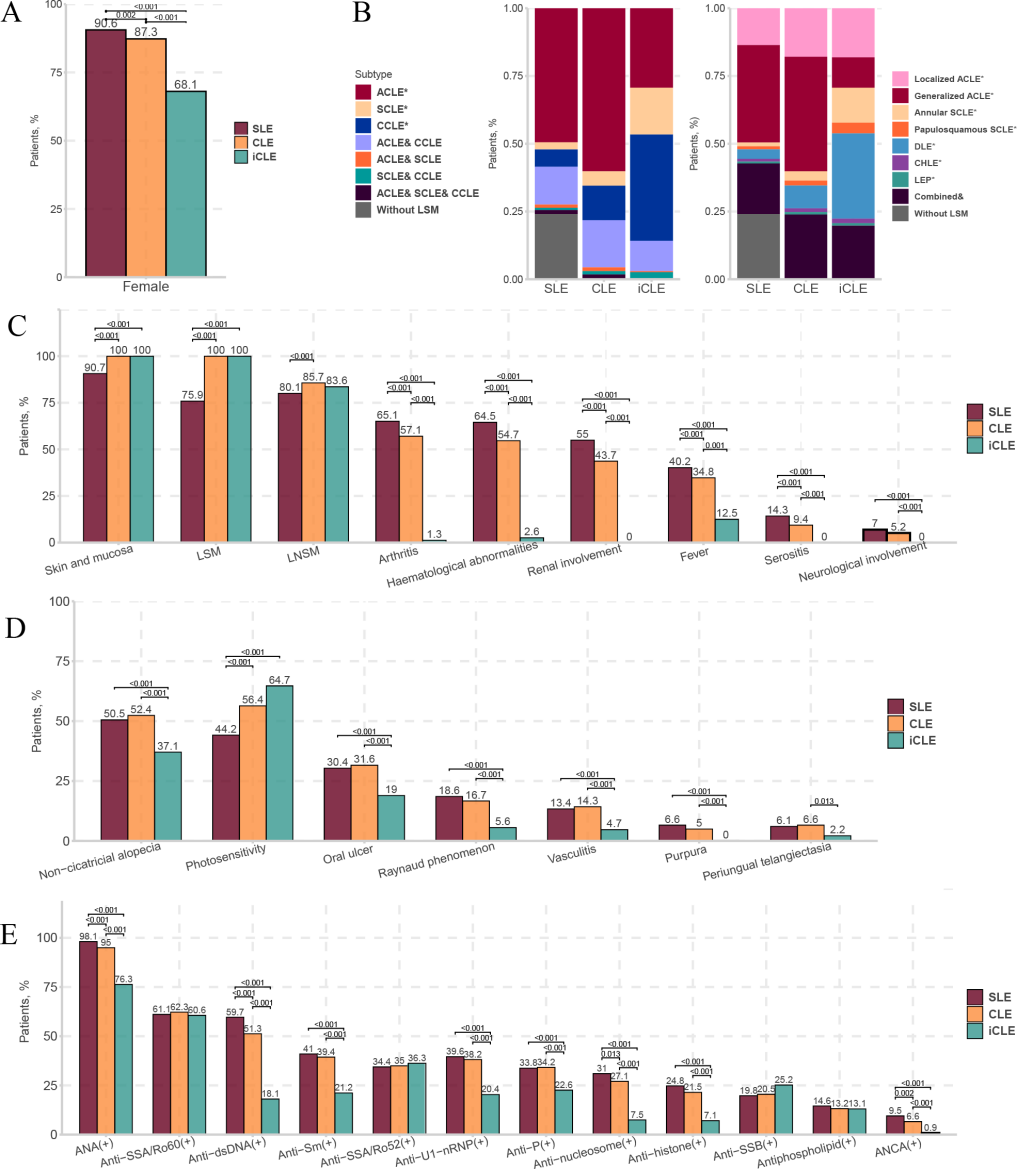
Overall demographic and clinical features in patients with SLE, CLE and iCLE and dissimilarities among groups. We present the female proportion (A), systemic involvements (C), LE-non-specific cutaneous manifestations (D) and autoantibodies (E) in patients with SLE, CLE and iCLE, with differences in pairwise comparison. (B) Distribution spectrum of patients with CLE subtypes together with their combined performances. Notably, only those with statistically significant differences are shown (D). For multiple comparisons, p value <Bonferroni-corrected p value, Bonferroni-corrected p value=0.017. *Only with the CLE subtype. ACLE, acute cutaneous lupus erythematosus; ANCA, antineutrophil cytoplasmic antibody; anti-P, anti-ribosomal P protein; anti-Sm, anti-Smith; anti-SSA/Ro52, anti-Sjögren’s syndrome-related antigen A/Ro52 kDa; anti-SSA/Ro60, anti-Sjögren’s syndrome-related antigen A/Ro60 kDa; anti-SSB, anti-Sjögren’s syndrome-related antigen B; anti-U1-nRNP, anti-U1 nuclear ribonucleoprotein; CCLE, chronic cutaneous lupus erythematosus; CHLE, chilblain lupus erythematosus; CLE, cutaneous lupus erythematosus; combined &, two or more subtypes of cutaneous lupus erythematosus concomitant occurrence; DLE, discoid lupus erythematosus; dsDNA, double-stranded DNA; iCLE, isolated cutaneous lupus erythematosus; LE, lupus erythematosus; LEP, lupus erythematosus profundus; LNSM, lupus erythematosus-non-specific cutaneous manifestation; LSM, lupus erythematosus-specific cutaneous manifestation; SCLE, subacute cutaneous lupus erythematosus.

In total, 2097 patients with LE were included: 1865 with SLE, 1648 with CLE and 232 with iCLE. Table 1 shows the demographic features of the study population and subgroups of patients. Among the three groups of patients with SLE, CLE and iCLE, the mean age of onset ranged from 27 years to 30 years, and the family history of LE ranged from 3.9% to 5.2%, with no significant intergroup differences. The proportion of women was significantly different between the groups, with SLE being the highest (90.6%), followed by CLE (87.3%) and iCLE (68.1%) (figure 2A).

**Table 1:** Demographic features of the population and subgroups of patients with LE

	n	Female, n (%)	Age at onset (year), mean (SD)	Course (month), mean (SD)	Family history of LE, n (%)
Overall patients	2097	1848 (88.1)	30.4 (12.4)	58.1 (67.1)	106 (5.1)
SLE	1865	1690 (90.6)	30.2 (12.2)	58.6 (67.1)	97 (5.2)
CLE	1648	1438 (87.3)	29.8 (12.1)	58.3 (66.4)	84 (5.1)
iCLE	232	158 (68.1)	32.1 (13.7)	53.6 (67.3)	9 (3.9)
SLE with LSM/CLE with SI	1416	1280 (90.4)	29.4 (11.8)	59.1 (66.3)	75 (5.3)
SLE without LSM	449	410 (91.3)	32.7 (13.0)	57.0 (69.4)	22 (4.9)
CLE without SI	232	158 (68.1)	32.1 (13.7)	53.6 (67.3)	9 (3.9)
ACLE	1330	1222 (91.9)	29.0 (11.6)	57.6 (64.2)	68 (5.1)
Localised ACLE	387	363 (93.8)	29.3 (11.7)	56.2 (60.1)	20 (5.2)
Generalised ACLE	943	859 (91.1)	28.8 (11.5)	58.1 (65.9)	48 (5.1)
SCLE	160	121 (75.6)	31.1 (12.9)	58.9 (68.1)	10 (6.2)
Annular SCLE	95	73 (76.8)	30.9 (13.2)	57.9 (70.3)	4 (4.2)
Papulosquamous SCLE	74	53 (71.6)	30.9 (12.1)	58.1 (62.5)	7 (9.5)
CCLE	546	437 (80.0)	31.0 (13.0)	65.1 (72.5)	35 (6.4)
DLE	311	224 (72.0)	32.4 (13.6)	64.8 (74.8)	19 (6.1)
CHLE	262	224 (85.5)	29.4 (12.1)	71.0 (73.9)	14 (5.3)
LEP	45	39 (86.7)	28.6 (10.3)	68.0 (63.2)	5 (11.1)

ACLE, acute cutaneous lupus erythematosus; CCLE, chronic cutaneous lupus erythematosus; CHLE, chilblain lupus erythematosus; CLE, cutaneous lupus erythematosus; DLE, discoid lupus erythematosus; iCLE, isolated cutaneous lupus erythematosus; LE, lupus erythematosus; LEP, lupus erythematosus profundus; LSM, lupus erythematosus-specific cutaneous manifestation; SCLE, subacute cutaneous lupus erythematosus; SI, systemic involvement.

Each group had a distinct and unique spectrum of LE-specific cutaneous manifestations. LE-specific cutaneous manifestations were observed in 75.9% of patients with SLE and ACLE in 66.2% of patients with SLE. Patients with iCLE had significantly more SCLE (mainly annular SCLE) and CCLE (mainly DLE) lesions but fewer ACLE (mainly generalised ACLE) lesions than patients with SLE (figure 2B and online supplemental figure 1). The SLE group had the highest proportion of systemic involvement and autoantibody positivity, followed by the CLE group and the iCLE group (figure 2C, E). The mucocutaneous system was the most frequently involved in patients with SLE (90.7%). Other systems included arthritis (65.1%), haematological abnormalities (64.5%), renal involvement (55.0%), fever (40.2%), serositis (14.3%) and neurological involvement (7.0%) (figure 2C). The iCLE group had lower proportion of most LE-non-specific cutaneous lesions but a significantly higher proportion of photosensitivity (64.7%) than the SLE group, suggesting that LE-non-specific cutaneous lesions may be associated with systemic involvement, while photosensitivity seems to be a mild sign (figure 2D and online supplemental table 1).

10.1136/lupus-2022-000819.supp1 Supplementary data

10.1136/lupus-2022-000819.supp2 Supplementary data

### Patients with SLE with and without LE-specific cutaneous manifestations, and patients with CLE with and without systemic involvement

**Figure 3:**
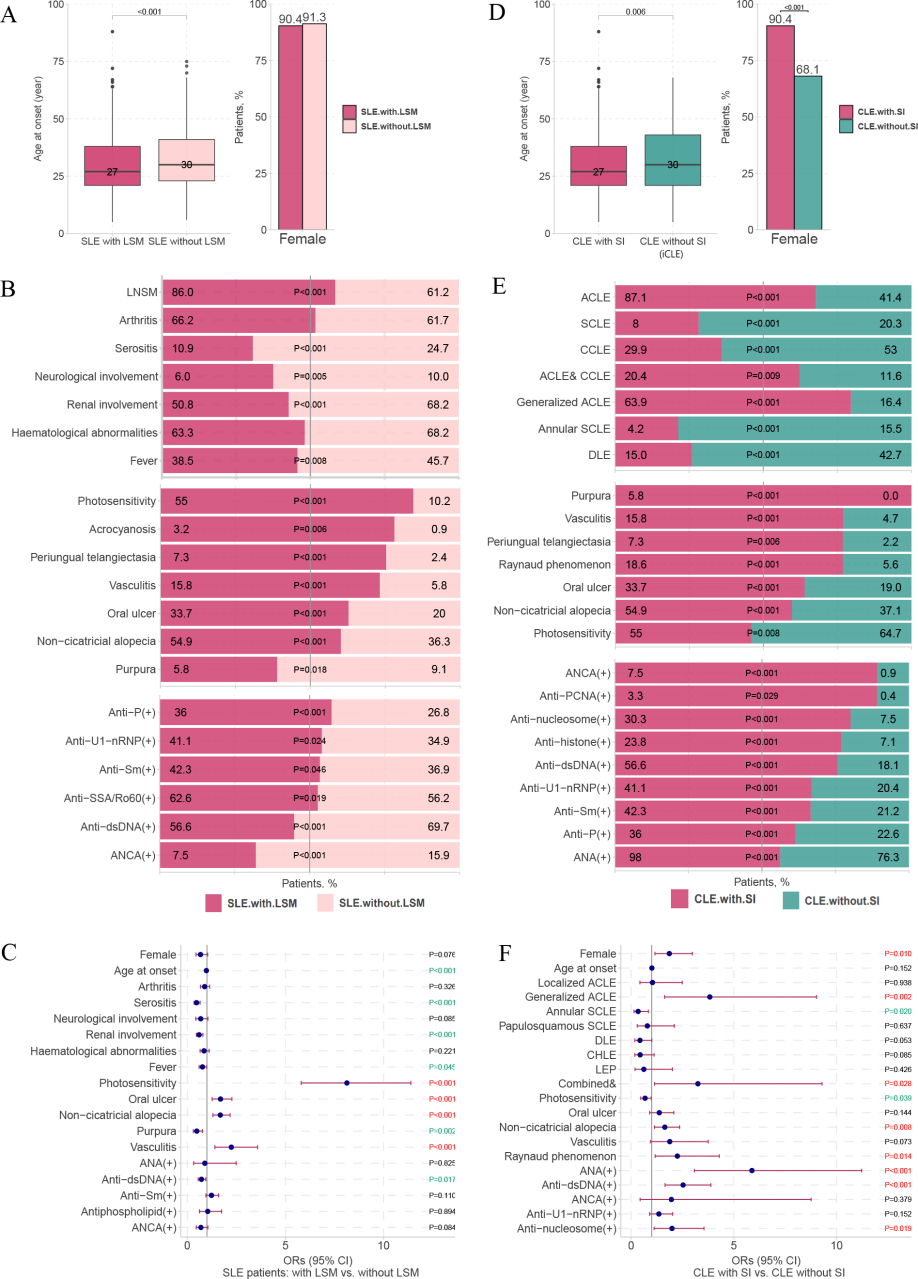
Comparisons between patients with SLE with and without LSMs, and comparisons between patients with CLE with and without SI (A) Comparisons of age at onset and female proportion between patients with SLE with and without LSMs. (B) Comparisons of SI, LNSMs and autoantibodies between patients with SLE with and without LSMs. (C) Multivariate logistic regression analysis between patients with SLE with and without LSMs. (D) Comparisons of age at onset and female proportion between patients with CLE with and without SI. (E) Comparisons of SI, LNSMs and immunological examinations between patients with CLE with and without SI. (F) Multivariate logistic regression analysis between patients with CLE with and without SI. Notably, only LNSMs and autoantibodies with statistically significant differences are shown (B, E). ACLE, acute cutaneous lupus erythematosus; ANCA, antineutrophil cytoplasmic antibody; anti-P, antiribosomal P protein; anti-Sm, anti-Smith; anti-SSA/Ro60, anti-Sjögren’s syndrome-related antigen A/Ro60 kDa; anti-U1-nRNP, anti-U1 nuclear ribonucleoprotein; CCLE, chronic cutaneous lupus erythematosus; CHLE, chilblain lupus erythematosus; CLE, cutaneous lupus erythematosus; combined &, two or more subtypes of cutaneous lupus erythematosus concomitant occurrence; DLE, discoid lupus erythematosus; dsDNA, double-stranded DNA; iCLE, isolated cutaneous lupus erythematosus; LE, lupus erythematosus; LEP, lupus erythematosus profundus; LNSM, lupus erythematosus-non-specific cutaneous manifestation; LSM, lupus erythematosus-specific cutaneous manifestation; PCNA, proliferating cell nuclear antigen antibody; SCLE, subacute cutaneous lupus erythematosus; SI, systemic involvement.

To further explore the relationship between systemic involvement and LE-specific cutaneous manifestations, we performed internal comparisons between the SLE and CLE groups. Compared with patients with SLE without LE-specific cutaneous manifestations, those with LE-specific cutaneous manifestations had an earlier age of onset (figure 3A) and less visceral involvement (serositis, neurological involvement, renal involvement and fever) but more photosensitivity and LE-non-specific cutaneous lesions (except purpura). In addition, patients with SLE with LE-specific cutaneous manifestations had more positive anti-Sjögren’s syndrome-related antigen A (SSA)/Ro60, anti-Smith (anti-Sm), anti-U1 nuclear ribonucleoprotein (anti-U1-nRNP) and antiribosomal P protein (anti-P) autoantibodies and fewer positive anti-double-stranded DNA (anti-dsDNA) and antineutrophil cytoplasmic antibody (ANCA) (figure 3B). The results of logistic regression confirmed that photosensitivity, oral ulcers, non-cicatricial alopecia and vasculitis tended to have higher concomitant LE-specific cutaneous manifestations, while older age at onset, serositis, renal disorders, purpura and anti-dsDNA (+) tended to have lower concomitant LE-specific cutaneous manifestations in patients with SLE (figure 3C).

Compared with patients with CLE without systemic involvement (ie, iCLE), the group with systemic involvement had an earlier onset age, higher frequency of women, ACLE (especially generalised ACLE) and more LE-non-specific cutaneous lesions and autoantibodies, but a lower frequency of SCLE (mainly annular SCLE), CCLE (mainly DLE) and photosensitivity (figure 3D, E). The results of logistic regression confirmed that female sex, generalised ACLE, combination with multiple CLE subtypes, non-cicatricial alopecia, Raynaud’s phenomenon, ANA (+), anti-dsDNA (+) and antinucleosome (+) tended to have higher concomitant systemic involvement, while annular SCLE and photosensitivity were less concomitant with systemic involvement in patients with CLE (figure 3F and online supplemental tables 2–4).

10.1136/lupus-2022-000819.supp3 Supplementary data

10.1136/lupus-2022-000819.supp4 Supplementary data

10.1136/lupus-2022-000819.supp5 Supplementary data

These results suggest that ACLE corresponds to a higher risk of systemic involvement; SCLE and CCLE correspond to a lower risk of systemic involvement; and that overall, the presence of LE-specific cutaneous manifestations is a protective signal for disease severity. In addition, photosensitivity has a high concomitant tendency with LE-specific cutaneous manifestations and is an independent protective factor for systemic involvement. Online supplemental figure 2 shows the association of photosensitivity with other clinical manifestations. The frequency of photosensitivity was highest in patients with both LE-specific and LE-non-specific cutaneous lesions, second in patients with only LE-specific cutaneous manifestations and the lowest in patients with only LE-non-specific cutaneous lesions (online supplemental figure 2B). The results of logistic regression confirmed that photosensitivity was an independent protective factor of multiple systemic involvements (except arthritis) and had a high concomitant trend with most LE-specific cutaneous manifestations and three LE-non-specific cutaneous lesions (oral ulcers, non-cicatricial alopecia and periungual telangiectasia) (online supplemental figure 2C, D further confirmed our findings about photosensitivity in figures 2D and 3C, F).

10.1136/lupus-2022-000819.supp6 Supplementary data

### Horizontal comparison between patients with different CLE subtypes

**Figure 4:**
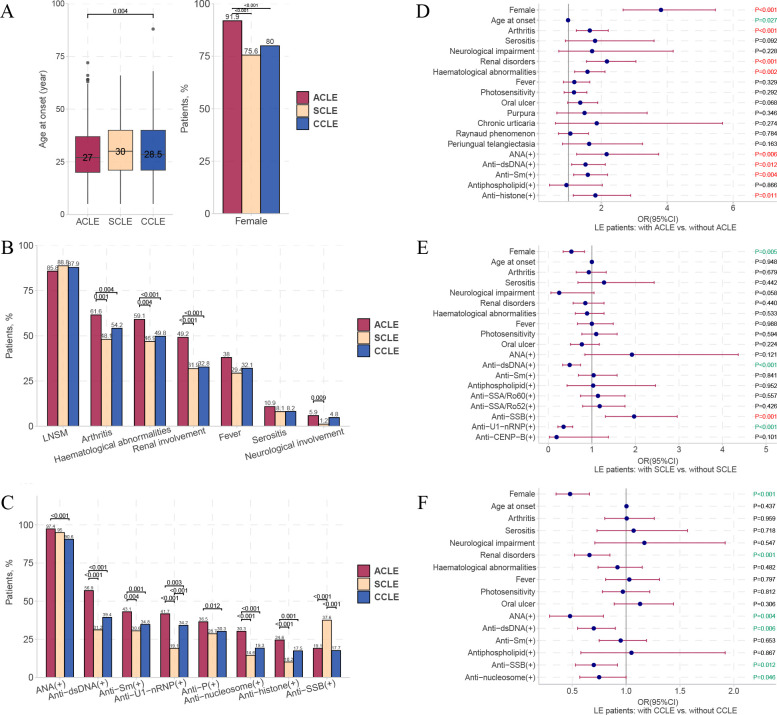
Clinical manifestations of patients with ACLE, SCLE and CCLE and dissimilarities among groups. Distribution of age at onset and female proportion (A); different systemic involvement (B); and autoantibodies (C) in patients with ACLE, SCLE and CCLE, with differences in pairwise comparison. Multivariate logistic regression analysis results between patients with and without ACLE (D), SCLE (E) and CCLE (F), respectively. Only autoantibodies with statistically significant differences are shown (C). For multiple comparisons, p value <Bonferroni-corrected p value, Bonferroni-corrected p value=0.017. ACLE, acute cutaneous lupus erythematosus; anti-P, antiribosomal P protein; anti-Sm, anti-Smith; anti-SSA/Ro52, anti-Sjögren’s syndrome-related antigen A/Ro52 kDa; anti-SSA/Ro60, anti-Sjögren’s-syndrome-related antigen A/Ro60 kDa; anti-SSB, anti-Sjögren’s syndrome-related antigen B; anti-U1-nRNP, anti-U1 nuclear ribonucleoprotein; CCLE, chronic cutaneous lupus erythematosus; dsDNA, double-stranded DNA; LE, lupus erythematosus; LNSM, lupus erythematosus-non-specific cutaneous manifestation; SCLE, subacute cutaneous lupus erythematosus.

The onset age of ACLE was significantly earlier than that of CCLE (figure 4A). Patients with ACLE had a significantly higher proportion of women (91.9%), incidence of systemic involvement (arthritis, haematological abnormalities, renal involvement and neurological involvement) and positive rates of multiple autoantibodies compared with patients with SCLE and CCLE, while these characteristics were not significantly different between patients with SCLE and CCLE (figure 4A–C). Except for the higher proportion of oral ulcers in patients with ACLE than in patients with SCLE, there were no significant differences in LE-non-specific cutaneous manifestations among the three groups (online supplemental table 5). The positive rate of anti-Sjögren’s syndrome-related antigen B (SSB) was specifically high in patients with SCLE (figure 4C). To explore the concomitant trends of each CLE subtype with other clinical features, we performed logistic regression on patients with LE with or without ACLE/SCLE/CCLE. ACLE had a higher concomitant tendency with female sex, arthritis, renal involvement, haematological abnormalities, ANA (+), anti-dsDNA (+), anti-Sm antibodies (+) and antihistone (+) (figure 4D). SCLE was more concomitant with anti-SSB antibodies (+) but less concomitant with female sex, anti-dsDNA (+) and anti-U1-nRNP antibodies (+) (figure 4E). CCLE had lower concomitance with women, renal disorders and ANA (+), anti-dsDNA (+), and anti-SSB (+) (figure 4F) (online supplemental tables 5–8).

10.1136/lupus-2022-000819.supp7 Supplementary data

10.1136/lupus-2022-000819.supp8 Supplementary data

10.1136/lupus-2022-000819.supp9 Supplementary data

10.1136/lupus-2022-000819.supp10 Supplementary data

### Internal comparison between patients with different CLE subtypes

**Figure 5:**
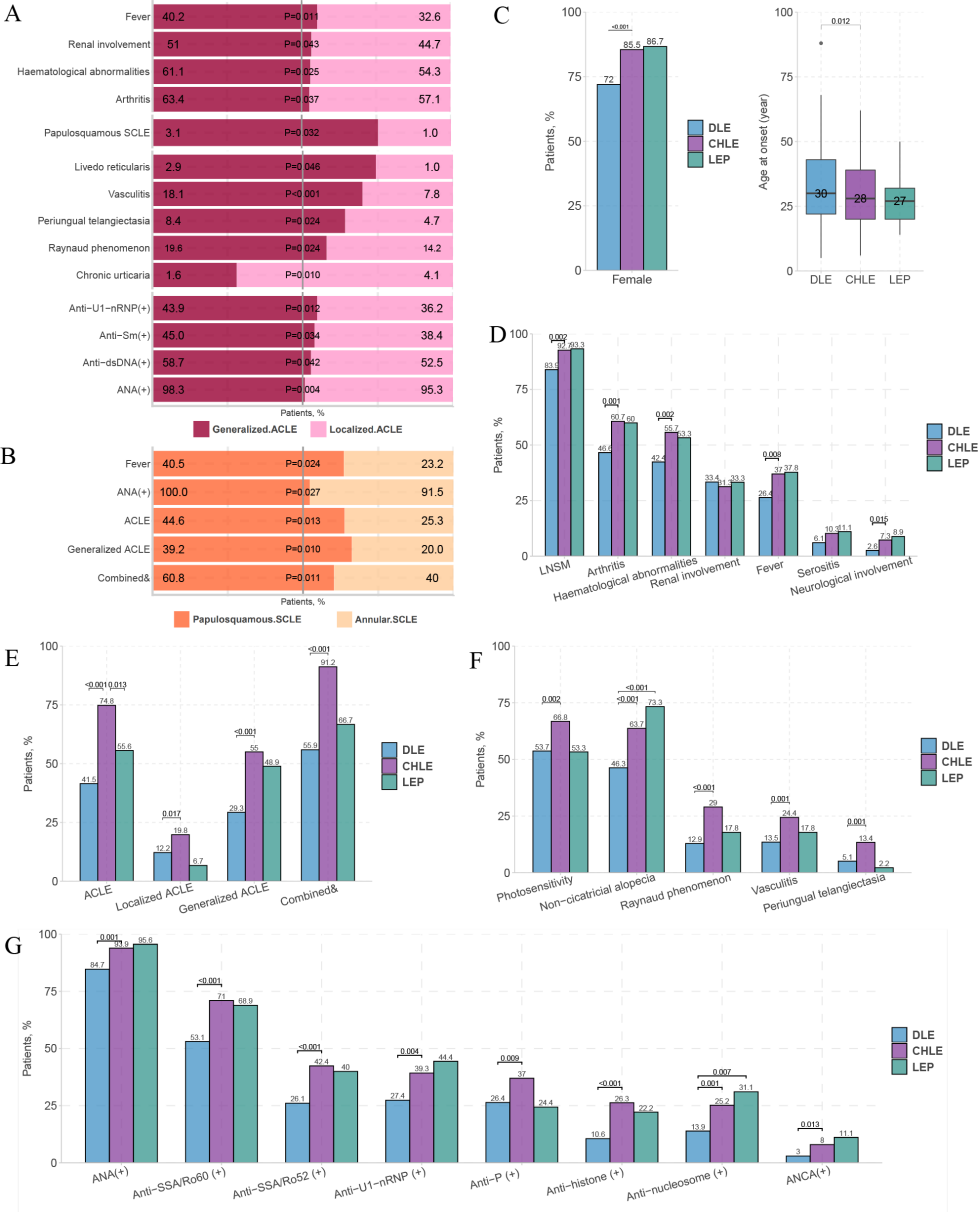
Dissimilarities in clinical manifestations of patients with each minor subtype of CLE. (A) Differences between patients with localised and generalised ACLE. (B) Differences between patients with annular and papulosquamous SCLE. Comparisons of age at onset and female proportion (C), systemic involvement (D); LSMs (E), LNSMs (F) and autoantibodies (G). Notably, only those with statistically significant differences are shown in (E–G). For multiple comparisons, p value <Bonferroni-corrected p value, Bonferroni-corrected p value=0.017. ACLE, acute cutaneous lupus erythematosus; ANCA, antineutrophil cytoplasmic antibody; anti-P, antiribosomal P protein; anti-Sm, anti-Smith; anti-SSA/Ro52, anti-Sjögren’s syndrome-related antigen A/Ro52 kDa; anti-SSA/Ro60, anti-Sjögren’s syndrome-related antigen A/Ro60 kDa; anti-U1-nRNP, anti-U1 nuclear ribonucleoprotein; CCLE, chronic cutaneous lupus erythematosus; CHLE, chilblain lupus erythematosus; CLE, cutaneous lupus erythematosus; combined &, two or more subtypes of cutaneous lupus erythematosus concomitant occurrence; DLE, discoid lupus erythematosus; dsDNA, double-stranded DNA; LE, lupus erythematosus; LEP, lupus erythematosus profundus; LNSM, lupus erythematosus-non-specific cutaneous manifestation; LSM, lupus erythematosus-specific cutaneous manifestation; SCLE, subacute cutaneous lupus erythematosus.

We conducted an in-depth internal comparison of the ACLE/SCLE/CCLE subtypes. In patients with ACLE, generalised ACLE was more associated with systemic involvement (fever, renal involvement, haematological abnormalities and arthritis), LE-non-specific cutaneous lesions (livedo reticularis, vasculitis, periungual telangiectasia and Raynaud’s phenomenon), positive autoantibodies (anti-U1-nRNP, anti-Sm, anti-dsDNA and ANA) and papulosquamous SCLE, whereas localised ACLE is more associated with chronic urticaria (figure 5A). Papulosquamous SCLE was more likely to be accompanied by fever, ANA (+) and combined types of CLE than annular SCLE; however, overall, there was no significant difference in most other clinical features between the two groups (figure 5B).

Our study included a large sample of patients with CCLE (n=546), including 311 patients with DLE, 262 patients with CHLE and 45 patients with LEP. We compared the three CCLE subtypes and found that most significant differences existed between CHLE and DLE. CHLE had a higher proportion of women and younger age of onset (figure 5C), a higher proportion of systemic involvement (arthritis, haematological abnormalities and fever), LE-non-specific cutaneous manifestations (photosensitivity, non-cicatricial alopecia, Raynaud’s phenomenon, vasculitis and periungual telangiectasia) and positive autoantibodies. In addition, LEP had a significantly higher proportion of non-cicatricial alopecia (73.3%) and antinucleosome (+) (31.1%) than DLE (figure 5D–G) (online supplemental table 9).

10.1136/lupus-2022-000819.supp11 Supplementary data

## Discussion

This was a cross-sectional multicentre study involving a large sample of patients with SLE, CLE and iCLE to analyse the demographic and clinical features of LE and the dissimilarities in different LE subtypes. We drew a panorama of lupus from Chinese patients, confirmed many previous conclusions or clinical experiences, and put forward some new conclusions. Notably, compared with another large Chinese lupus cohort, the Chinese SLE Treatment and Research (CSTAR) group,^24^ our research contains far more comprehensive lupus subtypes (CSTAR involved only patients with SLE) and conducted more in-depth and detailed research on cutaneous manifestations related to the different subtypes of lupus.

### What have we confirmed?

#### Different system involvement rates in patients with SLE

The mucocutaneous system was the most frequently involved organ in patients with SLE in our study (90.7%). The rates of other involved systems in patients with SLE were arthritis (65.1%)>haematological abnormality (64.5%)>renal involvement (55.0%)>serositis (14.3%)>neurological involvement (7.0%) (figure 2C). The most frequently involved systems in previous SLE studies were reported as cutaneous manifestations,^25–27^ arthritis^15 28^ and haematological abnormalities.^24 29^ Another large-scale study of patients with SLE in China (CSTAR)^24^ found similar system involvement rates (except for the cutaneous system) to those in our study. This difference might be explained by the insufficient data collection of cutaneous manifestations in most studies, especially those that did not include the ACR criteria. A few previous studies also presented cutaneous involvement rates similar to our research, reaching 90.1%^30^ and 90.7%.^25^

#### Relationship between systemic involvement, LE-specific cutaneous manifestations and LE-non-specific cutaneous lesions

Our results (figure 3B) and some previous studies^26 31–33^ showed that most LE-non-specific cutaneous lesions often appear simultaneously with LE-specific cutaneous manifestations. However, they have opposing implications for disease severity. Overall, patients with LE-specific cutaneous manifestations showed milder severity, whereas those with LE-non-specific lesions showed more severity (figure 2B). This is supported by previous findings that the majority of patients with CLE who develop SLE may have mild systemic involvement with primarily musculoskeletal manifestations.^7 32^ In particular, most previous studies and our study support the fact that patients with ACLE have the highest rate of systemic involvement, whereas patients with DLE have the lowest, with SCLE in the middle.^11 34 35^

LE-non-specific cutaneous lesions imply a high risk of systemic involvement, and disease activity has only been reported in a small number of studies with small sample sizes.^36 37^ Our study provides real-world evidence for this conclusion based on a large sample size, and we emphasise the warning role of purpura, vasculitis, periungual telangiectasia, Raynaud’s phenomenon, oral ulcers and non-cicatricial alopecia in systemic involvement (figures 2D and 3E).

### What have we proposed?

#### CLE and iCLE are two distinct disease states

The broad and narrow definitions of CLE have been disputed and lack a global consensus, which affects inconsistencies in the inclusion norms of scientific research and mismatches in results.^38^ Our results show that iCLE is a disease state independent of SLE and CLE, with a low female proportion (68.1%) and ANA positivity rate (76.3%), and is dominated by SCLE (20.3%) and CCLE (53.0%). CLE covers both the whole iCLE and part of the SLE, showing the transition characteristics between them. We call on scientific reports to emphasise the choice of a broad and narrow definition of CLE, as CLE and iCLE are distinct disease states.

#### Self-reported photosensitivity suggests a lower risk of systemic involvement

Almost all patients with LE would show clinical or histological evidence of aberrant photosensitivity when phototesting protocols are used, regardless of their self-reported photosensitive history or LE subtype.^39^ In the EUSCLE Core Set Questionnaire, photosensitivity was defined as ‘skin rash as a result of unusual reaction to sunlight, diagnosed by the patient’s history or physician’s observation’.^3^ In the face-to-face interviews, we asked patients whether sunlight exposure induced or aggravated their various types of rashes. Interestingly, though patients could not distinguish LE-specific or non-specific cutaneous lesions, and we indiscriminately included any related rashes, patients with LE-specific cutaneous manifestations reported a higher frequency of photosensitivity (figure 3C and online supplemental figure 2B). Moreover, patients with self-reported photosensitivity had lower frequency of multiple systemic involvements (figure 3F and online supplemental figure 2C, D). It has been noted that previous studies also reported that patients with LE-specific cutaneous manifestations have higher frequencies of photosensitivity.^11^

We would like to emphasise the difference between self-reported photosensitivity and broad photosensitivity. Broad photosensitivity refers to various symptoms or conditions (photodermatoses) caused or exacerbated by exposure to sunlight.^40^ Because of the delayed onset of photosensitive lupus manifestations, self-reported photosensitivity, usually manifesting as fast-response photodermatoses, is significantly less than the actual photosensitive reaction. Ultraviolet light is a well-recognised trigger for lupus;^39 40^ therefore, even if our results indicate a relatively benign course of disease in patients with self-reported photosensitivity (fast ultraviolet reaction), photoprotection is still essential, especially in patients without self-reported photosensitivity, by whom photoprotection is more likely to be ignored.^41^

#### Generalised ACLE is a more severe state than localised ACLE; CHLE is a more severe state than DLE

Large cohorts focusing on subtypes of LE-specific cutaneous manifestations (ACLE/SCLE/CCLE) are rare, and studies focusing on comparisons of more detailed subtypes (localised ACLE/generalised ACLE/annular SCLE/papulosquamous SCLE/DLE/CHLE/LEP) are lacking. Our study provided a comparison of detailed subtypes of LE-specific cutaneous manifestations in a larger sample.

ACLE (especially generalised ACLE) was the major LE-specific cutaneous manifestation of patients with SLE and CLE, while CCLE (especially DLE) was the major LE-specific cutaneous manifestation of patients with iCLE (figure 2B and online supplemental figure 1). We found that the risk of systemic involvement for patients with generalised ACLE tended to be higher than those with localised ACLE (figure 5A). The involvement rates of most visceral systems tended to be higher in papulosquamous SCLE, but this was not statistically significant when compared with annular SCLE in our study (figure 5B and online supplemental table 9).

According to some small sample cohorts, CHLE occurred in 6.0%–20.5% of patients with SLE, predominantly women, and approximately 18% of patients with CHLE progressed to SLE.^42^ In our study, CHLE was present in 12% of patients with SLE, with 85.5% of patients with CHLE being female. CHLE appeared to be a more severe condition than DLE, as it had a significantly higher proportion of systemic involvement, LE-non-specific cutaneous manifestations and positive autoantibodies.

#### Autoantibodies and CLE subtypes

Some positive autoantibodies (ANA, anti-dsDNA, anti-SSB, anti-U1-nRNP, anti-Sm, antihistone and antinucleosome) have a significantly higher or lower concomitant trend with ACLE, SCLE or CCLE (figure 4D–F). Notably, our results showed that dsDNA (+) had a higher co-occurrence with ACLE and a lower co-occurrence with SCLE and CCLE. We first report the association between dsDNA (+) and LE-specific cutaneous manifestations, which supplements the previous view that anti-dsDNA is an indicator of renal involvement.^43^ Besides, it has been claimed that UV-associated autoantibodies predominate in patients with SCLE: anti-SSA in 70%–80% and anti-SSB in 30%–40%,^4 11 44 45^ and we have further reported for the first time that anti-SSB antibodies have higher specific directivity than anti-SSA antibodies for SCLE lesions by the results of multivariate logistic regression between patients with and without SCLE (figure 4E). A study involving nine patients with CHLE found that CHLE was associated with anti-SSA/Ro antibodies.^46^ In our study, CHLE had significantly higher positive rates of anti-SSA/Ro60 (71%) and anti-SSA/Ro52 (42.4%) antibodies than DLE. In addition, we report for the first time that LEP is associated with a higher positive rate of antinucleosome antibodies (31.1%) than DLE.

### Limitations

This was not a population-based study, and we included more patients with SLE than patients with iCLE. However, a comparative analysis of different subgroups of LE is meaningful. Mucocutaneous features were assessed specifically in a Chinese cohort, and it is unclear if the results are generalisable to other ethnic groups with lupus.

## Conclusions

CLE and iCLE are two distinct disease states, and the selection of broad or narrow CLE definitions should be emphasised in scientific reports. LE-specific cutaneous manifestations and LE-non-specific cutaneous manifestations often appear simultaneously; LE-non-specific cutaneous lesions imply more severe severity, while photosensitivity and LE-specific cutaneous manifestations imply milder severity. Generalised ACLE appears to be a more severe state than localised ACLE, and CHLE appears to be more severe than DLE. Anti-SSB antibodies have a higher specific directivity than anti-SSA antibodies for SCLE lesions; anti-dsDNA antibodies have a higher co-occurrence with ACLE and a lower co-occurrence with SCLE and CCLE. Compared with DLE, CHLE has significantly higher positive rates of anti-SSA/Ro60 (71%) and anti-SSA/Ro52 (42.4%) antibodies, whereas LEP is associated with a higher positive rate of antinucleosome antibodies (31.1%). A comprehensive and in-depth understanding of the characteristics of different subgroups of patients with lupus will help stratify the management of patients, increase economic efficiency and improve prognoses.

10.1136/lupus-2022-000819.supp12 Supplementary data

## Data Availability

Data are available upon reasonable request. All data that support the findings of this study are available from the corresponding author on reasonable request.
