# Supplementary figures and images for: Panoramic view of clinical features of lupus erythematosus: a cross-sectional multicentre study from China
Source: Lupus Sci Med. 2023 Mar 20;10(1):e000819. doi: 10.1136/lupus-2022-000819 (PMC10030678; doi:10.1136/lupus-2022-000819)

B

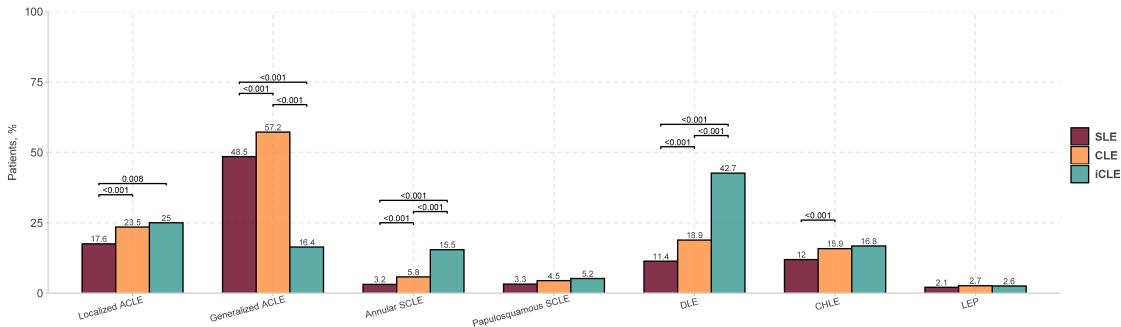

Supplement: Supplementary data [file lupus-2022-000819supp001.pdf]

A

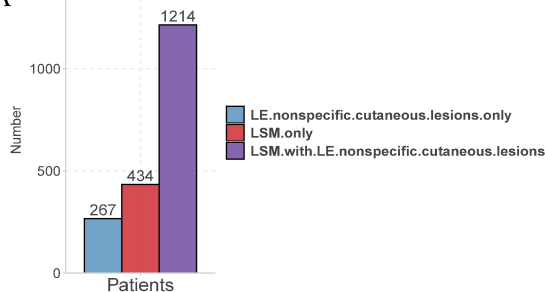

B

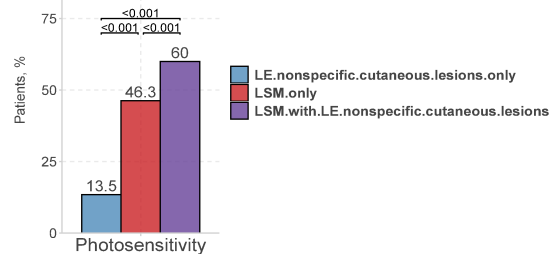

C

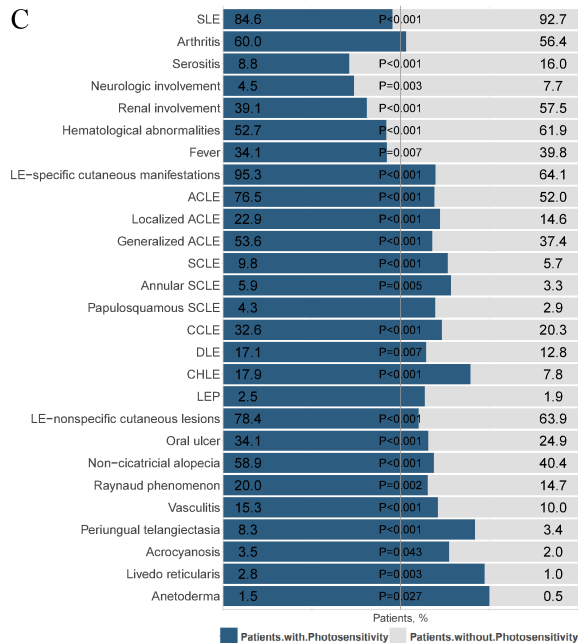

D

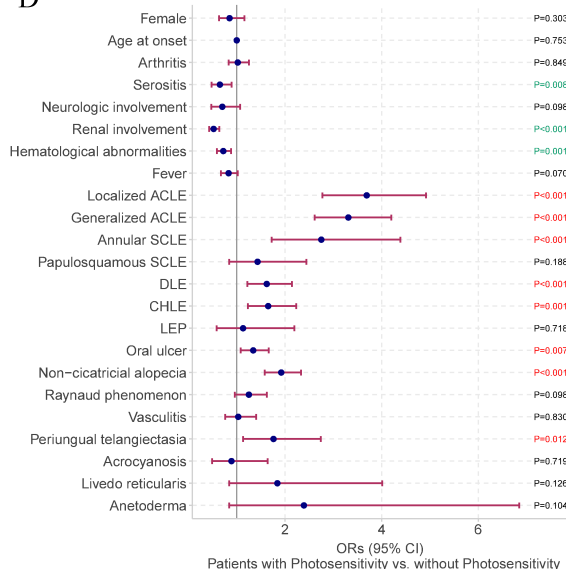

Supplement: Supplementary data [file lupus-2022-000819supp006.pdf]
